# OA10 When Q fever unmasks a thrombotic storm

**DOI:** 10.1093/rap/rkag113.010

**Published:** 2026-11-03

**Authors:** Omer Uludag, Serap Simsek Yavuz, Tuncay Gunduz, Berrin Umman, Bahar Artim Esen

**Affiliations:** Istanbul University, Istanbul Faculty of Medicine, Division of Rheumatology, Istanbul, Turkey; Istanbul University, Istanbul Faculty of Medicine, Department of Infectious Disease and Clinical Microbiology, Istanbul, Turkey; Istanbul University, Istanbul Faculty of Medicine, Department of Neurology, Istanbul, Turkey; Istanbul University, Istanbul Faculty of Medicine, Department of Cardiology, Istanbul, Turkey; Istanbul University, Istanbul Faculty of Medicine, Division of Rheumatology, Istanbul, Turkey

## Abstract

**Introduction:**

Antiphospholipid syndrome (APS) is an autoimmune disease characterized by arterial, venous, or microvascular thrombotic events, pregnancy morbidity, and non-thrombotic manifestations including heart valve disease, in the presence of persistent antiphospholipid antibody (aPL) positivity. While infections can cause transient aPL positivity, they can also act as thrombotic triggers or coexist with established APS, creating diagnostic and therapeutic challenges. Here, we present a case of culture-negative *Coxiella* endocarditis in a patient with systemic lupus erythematosus (SLE)-associated APS who presented with accelerated microvascular, valvular, and thromboembolic events.

**Case description:**

A 48-year-old man with a history of popliteal DVT presented with polyarthritis, photosensitivity, Raynaud’s phenomenon, and ANA positivity in 2009, which led to a diagnosis of SLE. Glucocorticoids and hydroxychloroquine were initiated. In 2012, he developed left-leg ulceration, intermittent diplopia, hallucinations, and personality change. Anticardiolipin (aCL) IgG was negative, IgM 34 MPLU, and anti-beta-2 glycoprotein I (aB2GPI) IgG/IgM 200/188. Warfarin and mycophenolate mofetil were added but taken irregularly.

In April 2023, he presented with fever, haemoptysis, and confusion. His haemoglobin was 10.3 g/dL, platelets 90,000/μL, creatinine 3.5 mg/dL, C-reactive protein 199 mg/L. Lupus anticoagulant (LA) was positive; aCL IgG/IgM 300/114 IU/mL, aB2GPI IgG/IgM 300/42 IU/mL, and urine protein-to-creatinine ratio 0.58 g/g. Chest CT showed ground-glass opacities compatible with alveolar haemorrhage. Transoesophageal echocardiography showed aortic valve vegetations measuring 18 mm and 10 mm. Brain MRI revealed multiple cerebral ischaemic lesions. Blood cultures were negative. He received broad-spectrum antibiotics, methylprednisolone 40 mg/day, hydroxychloroquine and warfarin. Rituximab was given after clinical improvement.

In November 2023, he presented with dyspnoea. Recurrent aortic vegetations, severe aortic regurgitation, and new cerebral ischaemic lesions were identified. His creatinine level was 1.5 mg/dL, C-reactive protein 19 mg/L and aCL IgG/IgM 96/11, aB2GPI IgG/IgM 143/90 IU/mL. The Coxiella burnetii phase I IgG titer was 1:32,768. The patient underwent a metallic aortic valve replacement and was started on a doxycycline and high dose hydroxychloroquine.

In June 2024, he presented with headache, confusion, and seizures. A subdural haematoma was detected. DSA showed an irregularity in the distal right middle cerebral artery, and echocardiography showed no new vegetation. Since *Coxiella* titres did not decline sufficiently, moxifloxacin replaced high-dose hydroxychloroquine; glucocorticoid and mycophenolate mofetil were added. The titre fell to 1:4096, seizures did not recur, and confusion improved.

**Discussion:**

This case highlights the complex interplay between Q fever and APS, as Q fever can mimic APS, induce transient aPL positivity and trigger thrombotic events in patients with established APS. In acute Q fever cohorts, LA and aCL positivity rates have been reported as high as 76% and 81%, respectively. However, these antibodies often disappear within three to six months of treatment and are not necessarily accompanied by thrombosis. Conversely, larger Q fever cohorts have shown that IgG aCL titres of > 100 GPLU and immunosuppression are associated with valvular vegetations or acute Q fever endocarditis. Furthermore, patients presenting with thrombosis and APS-like symptoms tend to have higher and more persistent titres.

In our patient, the chronology suggests that aPL positivity was not induced by infection alone. DVT occurred in 1994 and aPL positivity was first detected in 2012, which was years before *Coxiella* endocarditis was recognised. The presentation in April 2023, characterised by high-titre triple aPL positivity, thrombocytopenia, livedoid lesions, suspected diffuse alveolar haemorrhage, valve vegetations, and multifocal cerebral ischaemia, supported an established APS phenotype. Furthermore, despite a fall in the *Coxiella burnetii* phase I IgG titres to 1:4096 during the follow-up period, the aPL titres remained significantly high, including aCL IgG >300 IU/mL and aB2GPI IgG >300 IU/mL. However, *Coxiella* infection accounted for culture-negative endocarditis, recurrent vegetations and progressive aortic valve destruction. The later subdural haemorrhage and irregularity of the distal middle cerebral artery in angiography also raised the possibility of central nervous system vasculitis or vasculopathy related to infection, although this could not be confirmed. We therefore considered the infection to be a coexisting condition and possible second hit, rather than an alternative diagnosis. This case underscores the importance of balancing anticoagulation, antimicrobial treatment, and immunosuppression rather than treating thrombosis, infection, and inflammation as distinct entities.

**Key learning points:**

• Firstly, culture-negative endocarditis in patients with APS or SLE should not automatically be attributed to sterile antiphospholipid-related valve disease. Q fever should be considered if valvular vegetations recur, blood cultures are negative, inflammatory markers are high, embolisms occur, or valve destruction progresses. *Coxiella* serology can identify a treatable infection that might otherwise be difficult to detect due to a convincing autoimmune phenotype.

• Secondly, aPL positivity during an infection must be interpreted in a longitudinal context. Whilst Q fever can induce transient LA and aCL positivity, this does not exclude a diagnosis of APS. The presence of previous thrombosis, pre-existing aB2GPI positivity, a persistently high aPL titre despite falling *Coxiella* titres and compatible clinical manifestations all support the diagnosis of established APS rather than infection-induced aPL alone.

• Thirdly, infection and APS should not always be viewed as mutually exclusive diagnoses. In this case, *Coxiella* endocarditis explained the culture-negative endocarditis, the recurrent vegetations, and the progressive destruction of the aortic valve. Meanwhile, the long-standing thrombotic history and the persistently high titres of aPL supported the diagnosis of underlying APS. Therefore, infection was considered a possible “second hit” for accelerated thrombotic events.

• Fourthly, in SLE-associated APS and infection, neurological complications require a broad differential diagnosis. Cardioembolism, APS-related microvascular disease, haemorrhage associated with anticoagulation, accelerated vascular disease and infection-related central nervous system vasculitis or vasculopathy may overlap.

• In practice, it is important to balance anticoagulation, antimicrobial therapy, and immunosuppression dynamically through multidisciplinary assessment. Antimicrobial therapy for Q fever endocarditis requires prolonged treatment and close monitoring of the serological response. Meanwhile, immunosuppressive therapy must be carefully titrated to control autoimmune disease activity without exacerbating the infection. Regular collaboration between rheumatology, infectious diseases, cardiology, and neurology teams is essential to reassess disease activity, treatment response and emerging complications, and to allow for timely adjustments to therapy.

